# Are the skin scar characteristics and closure of the parietal peritoneum associated with pelvic adhesions?

**DOI:** 10.4274/tjod.55491

**Published:** 2018-03-29

**Authors:** Numan Çim, Erkan Elçi, Gülhan Güneş Elçi, Necat Almalı, Recep Yıldızhan

**Affiliations:** 1 Van Yüzüncü Yıl University Faculty of Medicine, Department of Gynecology and Obstetrics, Van, Turkey; 2 University of Health Sciences, Van Training and Research Hospital, Clinic of Gynecology and Obstetrics, Van, Turkey; 3 Van Yüzüncü Yıl University Faculty of Medicine, Department of General Surgery, Van, Turkey

**Keywords:** Skin scar, pelvic adhesion, closure of parietal peritoneum

## Abstract

**Objective:**

To assess whether the abdominal scar characteristics and closure of the peritoneum were associated with pelvic adhesions.

**Materials and Methods:**

Patients who had undergone cesarean section between December 2015 and February 2016 were assessed prospectively in terms of age, gravida, body mass index, number of living children, number of cesarean sections, time passed since the last cesarean section, closure status of the peritoneum in the last cesarean section, presence of other diseases, smoking status, location of incision in the abdomen (medial, pfannenstiel) scar dimensions (length, width), scar status with respect to skin (hypertrophic, flat, depressive), scar color [color change/no color change (hyperpigmented/hypopigmented)], adhesion of bowel-omentum-uterus, omentum-anterior abdominal wall, uterus-anterior abdominal wall, uterus-bladder, bladder-anterior abdominal wall, fixed uterus, and uterus-omentum-anterior abdominal wall in abdominal exploration.

**Results:**

One hundred five pregnant women who had undergone previous ceserean section surgery by the same physician, were at least in their 30th gestational week, had surgery notes about their previous operation, and had no chronic diseases were included in the study. Age, gravida, body mass index, number of children, number of cesarean sections, time passed since the previous cesarean section, closure/non-closure of peritoneum in the previous cesarean section, and smoking status had no effect on pelvic adhesions. Intraabdominal adhesion was not found to be associated with scar length [odds ratio (OR): 1.54, 95% confidence interval (CI): 1.1-2.2; p=0.02], depressive scar (OR: 9.3, 95% CI: 3.2-27.2; p<0.001), or hypopigmented scar [OR: 0.01, 95% CI: 0.003-0.11; p<0.001].

**Conclusion:**

Adhesions following surgical operations are of great importance due to complications for the patient, complications in relaparotomy, and high costs. Depressive and hypopigmented abdominal scars may be associated with pelvic adhesions. We believe that closure or non-closure of the parietal peritoneum is not associated with pelvic adhesions.

**PRECIS:** Although depressive and hypopigmented abdominal scars are associated with pelvic adhesion, there is no association between peritoneal closure and pelvic adhesions.

## Introduction

In spite of advancements in surgical techniques and the emergence of substances that prevent adhesions, pelvic adhesions continue to be a problem for the patient and the physician. As in every surgical branch, pelvic adhesions also cause many long-term problems in gynecologic and obstetric operations. Major problems caused by pelvic adhesions include various complications such as organ damage in future operations, intestinal obstruction, and chronic pelvic pain^(1)^. The aim of this study was to prospectively investigate whether surgical scar characteristics and closure/non-closure of the peritoneum in the previous operation were associated with pelvic adhesions.

## Materials and Methods

Pregnant women at the 30^th^ gestational week or over who underwent cesarean section (CS) between December 2015 and February 2016 in the Van İpekyolu Maternity and Children’s Hospital and Yüzüncü Yıl University Faculty of Medicine, Department of Gynecology and Obstetrics, were included in the study. The study was approved by the University of Health Sciences, Van Training and Research Hospital Local Ethics Committee (approval number: 21.04.2015-2015/3). Informed consent forms were filled out by all participants. The participants had previously undergone at least one other CS by the same surgeon. The scar location in the previous CS, scar dimensions (length, width), scar’s status with respect to skin (hypertrophic, flat, depressive), scar color [color change/no color change (hyperpigmented/hypopigmented)] were noted retrospectively for the participants. Whether the parietal peritoneum was closed and pelvic adhesions during the operation were examined using the records of the previous CS. Those who did not have surgical operation notes, received scar revision in the previous operation, had chronic diseases (diabetes mellitus, inflammatory bowel diseases, Familial Mediterranean fever, endometriosis), had chronic steroid use, and low-molecular-weight heparin use were excluded from the study. 

In compliance with the literature, 4 different scores were used to assess abdominal adhesions in the most recent CS. No adhesions was scored as 0, filmy adhesions needing blunt dissection were scored as 1, strong adhesions requiring sharp dissection were scored as 2, and very strong vascularized adhesions that required sharp dissection and hardly preventable damage were scored as 3^(2)^.

### Cesarean technique

The most current and the previous cesarean operations performed by the surgeons in the study involved cutting the skin and the subcutaneous tissue transversely at 2 cm above the pubic bone (Pfannenstiel incision) under general and spinal anesthesia, then blunt dissecting the rectus sheath with a finger, and entering the abdomen. After cutting the lower segment of the uterus transversely (Kehr incision), the infant was delivered. The uterus incision was closed in a continuous interlocking manner in a single layer and the endometrium, myometrium, and perimetrium layers facing each other (entering from the perimetrium and exiting from endometrium boundary; entering from endometrium boundary and exiting from perimetrium boundary) using absorbable 1-0 Vicryl suture (Ethicon, Piscataway, NJ). The abdomen was cleaned (amnion fluid and clots were removed), and checked for bleeding. The peritoneum was closed with 2-0 Monocryl (not closed in some cases). The rectus fascia was closed in a continuous manner using a 1-0 Vicryl suture (Ethicon, Piscataway, NJ). After the subcutaneous bleeding inspection, the skin was closed with a 2-0 Monocryl suture subcutaneously. Prophylactic antibiotic was given intraoperatively to all patients after delivery of the fetus (urgent, elective) as 1 g intravenous cefazolin sodium.

### Statistical Analysis

Descriptive data are presented as mean ± standard deviation, median, and ratio. The non-parametric Mann-Whitney U test was used to compare data between the groups. p<0.05 was considered to be statistically significant. SPSS 22.0 was used for data analysis. Logistic regression analysis was used to determine significant predictors of intraabdominal adhesions. In the logistic regression, intraabdominal adhesion scores were used as dependent variables, and age, gestational week, body mass index (BMI), smoking, parity, the number of previous CS, time passed since the previous CS, the presence of peritonization in the previous CS, and skin scar (length, width, color, pigmentation) were used as independent variables. The data were stored in STATA 13.0 (Stata Corporation, Texas, USA) and the entire analysis was performed using this system. P<0.05 was accepted as the level of statistical significance.

## Results

The demographic characteristics of the patients including age, gestational week, BMI, number of CSs, and time passed since the previous CS can be seen in Table 1 as mean values and standard deviation. Age, gestational week, BMI, smoking, parity, the number of previous CSs, time passed since the previous CS, and the presence of peritonization in the previous CS had no effect on the probability of intraabdominal adhesions (Table 2). There was a significant association between scar length [odds ratio (OR): 1.54, 95% confidence interval (CI): 1.1-2.2; p=0.02], depressed scar (OR: 9.3, 95% CI: 3.2-27.2; p<0.001), hypopigmented scar (OR: 0.01, 95% CI: 0.003-0.11; p<0.001) and the amount of intraabdominal adhesions. No association was found between scar color and the amount of intraabdominal adhesions.

## Discussion

The incidence of adhesions following surgical operations is reported to be 93%, which is a very high level^(3)^. Such adhesions occur in one tenth or more of the patient’s abdominal cavity^(4)^. Adhesions following surgical operations may lead to infertility, organ damage due to adhesions in future operations, intestinal obstruction, and chronic pelvic pain^(5)^. Higher numbers of CSs received by patients leads to more frequent organ damage in following relaparotomies. The annual cost incurred due to complications caused by pelvic adhesions is estimated to be 1.2 million dollars in the United States^(6)^. Independent from bleeding, the physiopathology of intraabdominal adhesions following surgical operations is reported to be induced by fibrin clots due to fibrinogen activated by the tissue factor or more specifically, the “fibrin gel matrix”^(7)^. Fibrinogen emerging from surfaces damaged during the surgical operation is a soluble protein, which forms fibrin monomers by reacting with thrombin and polymerizes. Fibrin polymers must be removed when they emerge because they are initially soluble. If they remain for prolonged periods, they contact with certain coagulation factors such as Factor XIIIa, become insoluble, and form a fibrin gel matrix^(8)^. Damage in the peritoneum associated with trauma and ischemia induces a quick response and the damaged regions are closed by neutrophils within four hours. Complete healing after constant reactions occurs within approximately one week^(9)^. As can be understood from the information given above, the formation of adhesion begins with the release of tissue factors. For this reason, the size of the scar may be associated with intraabdominal adhesions. We found a relationship between cesarean incision and intraabdominal adhesion (OR: 1.54, 95% CI: 1.1-2.2; p=0.02). 

The hypothesis that peritoneal fibrinolytic activity plays an important role in the pathophysiology of the dissociation of adhesions has been suggested^(7)^. Tissue plasminogen activator (tPA) in mesothelial cells is a significant natural defense against the formation of adhesions following surgical operations. Active fibrinolysis enzymes, which emerge from inactive plasminogen via tPA, turn the fibrin gel matrix into fibrin destruction products that have no effect on the formation of adhesions^(7)^. If local fibrinolysis is sufficient, fibrinous adhesions are lysed. If it is not sufficient, connective tissue formation and adhesion development may occur^(9)^. It was observed in many studies that closure of the parietal peritoneum increased adhesions in gynecologic operations,^(10)^ general surgical operations,^(11)^ and animal experiments^(12)^. Based on the above data, the parietal peritoneum is routinely closed in gynecologic and obstetric operations^(10,13)^. However, some other studies reported that, unlike other abdominal operations, closure of the peritoneum decreased pelvic adhesions in pregnant women^(14)^. However, with respect to the significance of the fibrinolytic activity in the pathophysiology explained above, amnion fluid was found to show significant fibrinolytic activity after the 37^th^ gestational week^(15)^. Myers  and Bennett^(16) ^and Roset et al.^(17) ^reported that the closure of the parietal peritoneum reduced adhesions in pregnant women. In the present study, we found intraabdominal adhesions were not affected by whether the parietal peritoneum was closed (OR: 0.74, 95% CI: 0.34-1.61, p=0.45). The healing phases of skin scars includes the inflammatory phase (including the injury and prevents infection), the proliferative phase (granulation of macrophages, proliferative degeneration, and characterized by epithelial tissue), and the remodeling phase (regulation of the extracellular matrix), which is a long process. Considering the significant points of the molecular biology behind the healing of scars, the factor that is effective at the molecular level is transforming growth factor-beta (TGF-β)^(18)^. In adults, TGF-β and receptors are observed to be evidently active in scar tissue and involved in scar formation at the site of injury. However, TGF-β expression is temporary in the fetus and does not form scar tissue^(19)^. Additionally, fibroblasts were observed to synthesize proteins involved in continuous TGF-β signal transduction in both hypertrophic scars and keloids^(20,21,22,23,24)^. The number of studies on the relationship between scar tissue and intraabdominal adhesions is limited. However, Salim et al.^(25) ^found that, among all abdominal scar characteristics, only depressive scars were associated with an increase in number and severity of evident adhesion incidence. Also, the incidence of frozen pelvis was found to increase by almost 12 times in women with depressive scars compared with those without depressive scars. Similar to many other researchers, Ferreira et al.^(26) ^reported that hormonal, immunologic, genetic, and tissue growth factors played significant roles in scar development. Nissen et al.^(27) ^showed that filmy intraabdominal adhesions, excessive fibrovascular structures, and depressive scars led to hypertrophic scars and might be affected by tensile strength. In this study, we found that hypopigmentation and depression of scars were associated in with intraabdominal adhesions (p<0.001, p<0.001). 

It was reported in a previous study that there was no significant difference between women who underwent CS only once and women who underwent CS two or three times in terms of intraabdominal adhesion incidence^(25)^. According to other studies, no difference was reported between women with multiple abdominal incisions and women with a single abdominal incision in terms of intraabdominal adhesions^(28,29)^. Similarly, we observed no statically significant difference between women with different numbers of CSs in terms of abdominal adhesions (OR: 1.09, 95% CI: 0.52-2.26; p=0.80).

### Study Limitations

A limitation of our study is the small number of patients. Another limitation is that we did not emphasize whether the first cesarean operation of patients was performed as an emergency or electively.

## Conclusion

Adhesions following surgical operations are of great importance due to complications for the patient (e.g., infertility, chronic pelvic pain), and complications in relaparotomy, and high costs. Depressive and hypopigmented abdominal scars provide important information for preoperative prediction of pelvic adhesions. We found that closure or non-closure of the parietal peritoneum was not associated with pelvic adhesions. We believe that more comprehensive studies are required to explain the effect of factors involved in pelvic adhesions.

## Figures and Tables

**Table 1:**
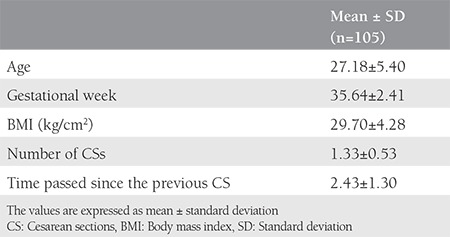
Mean values of the demographic characteristics of the participants

**Table 2:**
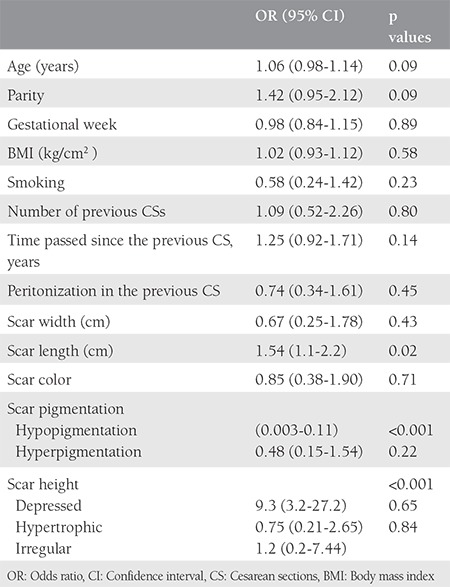
Association between the amount of intraabdominal adhesion and its possible predictors
